# A research and development (R&D) roadmap for broadly protective coronavirus vaccines: A pandemic preparedness strategy

**DOI:** 10.1016/j.vaccine.2023.02.032

**Published:** 2023-03-24

**Authors:** Kristine A. Moore, Tabitha Leighton, Julia T. Ostrowsky, Cory J. Anderson, Richard N. Danila, Angela K. Ulrich, Eve M. Lackritz, Angela J. Mehr, Ralph S. Baric, Norman W. Baylor, Bruce G. Gellin, Jennifer L. Gordon, Florian Krammer, Stanley Perlman, Helen V. Rees, Melanie Saville, Charlotte L. Weller, Michael T. Osterholm, The Coronavirus Vaccines R&D Roadmap Taskforce

**Affiliations:** a Center for Infectious Disease Research and Policy, University of Minnesota, Minneapolis, Minnesota, USA; b Center for Infectious Disease Research and Policy, C315 Mayo Memorial Building, MMC 263, 420 Delaware Street, SE, Minneapolis, Minnesota 55455, USA; c Minnesota Department of Health, St. Paul, Minnesota, USA; d University of North Carolina, Chapel Hill, North Carolina, USA; e Biologics Consulting Group, Inc., Alexandria, Virginia, USA; f The Rockefeller Foundation, Washington, DC, USA; g National Institute of Allergy and Infectious Diseases, National Institutes of Health, Bethesda, Maryland, USA; h Department of Microbiology, Department of Pathology, Molecular and Cell-Based Medicine, and Center for Vaccine Research and Pandemic Preparedness (C-VaRPP), Icahn School of Medicine at Mount Sinai, New York City, New York, USA; i University of Iowa, Iowa City, Iowa, USA; j Wits RHI, Faculty of Health Sciences, University of the Witwatersrand, Johannesburg, South Africa; k Coalition for Epidemic Preparedness Innovations, London, United Kingdom; l Wellcome Trust, London, United Kingdom

**Keywords:** Coronavirus, Pandemic preparedness, COVID-19 vaccines, Coronavirus vaccines, Broadly protective coronavirus vaccines, Roadmap, Vaccine research

## Abstract

Broadly protective coronavirus vaccines are an important tool for protecting against future SARS-CoV-2 variants and could play a critical role in mitigating the impact of future outbreaks or pandemics caused by novel coronaviruses. The Coronavirus Vaccines Research and Development (R&D) Roadmap (CVR) is aimed at promoting the development of such vaccines. The CVR, funded by the Bill & Melinda Gates Foundation and The Rockefeller Foundation, was generated through a collaborative and iterative process, which was led by the Center for Infectious Disease Research and Policy (CIDRAP) at the University of Minnesota and involved 50 international subject matter experts and recognized leaders in the field. This report summarizes the major issues and areas of research outlined in the CVR and identifies high-priority milestones. The CVR covers a 6-year timeframe and is organized into five topic areas: virology, immunology, vaccinology, animal and human infection models, and policy and finance. Included in each topic area are key barriers, gaps, strategic goals, milestones, and additional R&D priorities. The roadmap includes 20 goals and 86 R&D milestones, 26 of which are ranked as high priority. By identifying key issues, and milestones for addressing them, the CVR provides a framework to guide funding and research campaigns that promote the development of broadly protective coronavirus vaccines.

## 1 Introduction

### 1.1 The coronavirus pandemic threat

For years before the COVID-19 pandemic created global havoc, experts around the world warned about the potential of a catastrophic pandemic, most likely to be caused by a novel reassortant strain of influenza. A number of experts, however, also noted the potential for a novel coronavirus to cause a pandemic, particularly following the emergence of two highly pathogenic coronaviruses—severe acute respiratory syndrome coronavirus (SARS-CoV) and Middle East respiratory syndrome coronavirus (MERS-CoV)—coupled with identifying high-risk coronavirus strains circulating in animals [1], [2], [3], [4], [5], [6]. Severe acute respiratory syndrome coronavirus-2 (SARS-CoV-2), although less virulent than SARS-CoV or MERS-CoV, is much more transmissible between humans and, therefore, spread rapidly around the world in early 2020 to precipitate the COVID-19 pandemic. By the end of 2022, the World Health Organization (WHO) had recorded more than 650 million COVID-19 cases and 6.6 million deaths worldwide [7], with many survivors suffering long-term health effects [8]. Since the ancestral strain of SARS-CoV-2 emerged in late 2019, the virus has demonstrated the ability to evolve rapidly and mutate toward greater viral fitness, enhanced transmission kinetics, and immune escape [9], [10].

### 1.2 Vaccines for addressing pandemic threats

A primary strategy for mitigating or protecting against infectious diseases is vaccination. Several initiatives around the world, including Operation Warp Speed in the United States; COVAX led by the Coalition for Epidemic Preparedness Innovations (CEPI), WHO, and Gavi, the Vaccine Alliance; and other efforts in the public and private sectors, were able to develop, advance, and deploy safe and effective vaccines against SARS-CoV-2 on a faster timeline than ever before [11], [12]. This remarkable achievement was possible in part because of important foundational research into structure-based antigen design and novel vaccine platforms conducted in the decades before the pandemic [13], [14]. Despite the successful development and authorization of efficacious vaccines, uneven vaccine distribution and uptake has occurred globally. Additionally, immunity from neutralizing antibodies generated by existing vaccines is relatively short-lived and does not confer sterilizing immunity, which allows for ongoing transmission [15], [16]. Furthermore, SARS-CoV-2 has continued to circulate and evolve, resulting in SARS-CoV-2 variants of concern that can evade immune protection from infection or vaccination. Finally, given recent experience, other novel coronaviruses with pandemic potential will likely emerge from animal reservoirs [1], [4], and our current coronavirus vaccines will provide minimal or no protection against them.

One approach to ensure that vaccines are available quickly for mitigating a new coronavirus threat is to have rapid-response capabilities in place for just-in-time vaccine development, manufacture, and distribution. CEPI, for example, has promoted the concept that rather than preparing vaccines in advance of a threat, vaccines should be ready for initial authorization and manufacturing at scale within 100 days after the next pandemic threat is recognized [17]. This strategy has a broader scope than just coronaviruses and relies on systematically developing and evaluating “prototype vaccines” for a variety of known pathogens in different virus families, including the Coronaviridae family. Another approach that is more specific for coronaviruses is to develop broadly protective, or even universal, vaccines against a range of coronavirus species, and to ensure that such vaccines are available either for routine use (such as for those at high-risk of exposure) or can be stockpiled for rapid deployment, with a plan for scale-up as needed if a novel coronavirus with pandemic potential emerges [18], [19], [20].

### 1.3 Strategies for developing broadly protective coronavirus vaccines

Coronaviruses are enveloped, single-stranded ribonucleic acid (RNA) viruses that include four genera: alphacoronaviruses, betacoronaviruses, gammacoronaviruses, and deltacoronaviruses. Betacoronaviruses are of greatest concern, since this genus includes SARS-CoV, MERS-CoV, and SARS-CoV-2. The potential for additional betacoronaviruses or viruses from other coronavirus genera to spill over to humans and cause significant disease is unknown, but the risk cannot be ignored.

**Fig. 1:**
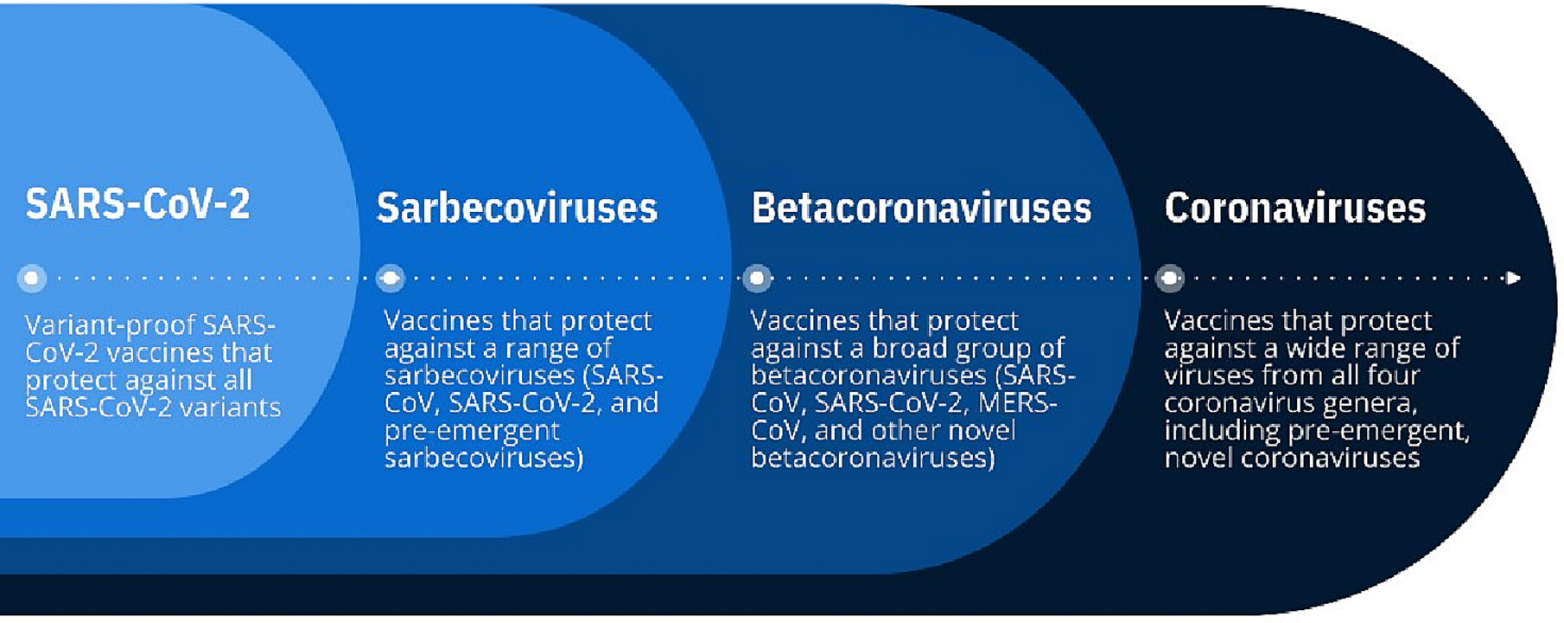
A tiered approach to developing broadly protective coronavirus vaccines.

Given this backdrop, one strategy for developing broadly protective coronavirus vaccines is to apply a tiered approach, beginning with the most urgent threats and then progressing over time to broader protection, encompassing more coronavirus genera and subgenera as new scientific information becomes available (Fig. 1). This strategy could focus initially on creating “variant-proof” SARS-CoV-2 vaccines that would protect against all SARS-CoV-2 lineages and sub-lineages. A second tier would be to create vaccines that protect against a wide range of sarbecoviruses, including SARS-CoV and SARS-CoV-2 variants and other novel coronaviruses in the sarbecovirus subgenus. A third tier would be to create vaccines that protect against a broad group of betacoronaviruses, including SARS-CoV, SARS-CoV-2, MERS-CoV, and other pre-emergent betacoronaviruses identified from zoonotic reservoirs that have the potential to spill over into humans. A final tier would be to develop vaccines that protect against a wide range of viruses from all four coronavirus genera, including the milder “common cold” species and any pre-emergent, novel coronaviruses with pandemic potential; such vaccines are also referred to as “universal” or “pan-coronavirus” vaccines. Within all tiers, the primary goal of vaccination would be to prevent severe disease and death, with prevention of transmission being an optimal goal.

A key lesson learned from the rollout of COVID-19 vaccines is that significant inequities in access to approved vaccines occurred, particularly among low- and middle-income countries (LMICs). Factors that contributed to this disparity included the consideration of national interests in the face of limited supply, generation of products with stringent technical requirements (such as cold-chain requirements), and existing global inequities in public-health capacity, infrastructure, financing, technology transfer, and manufacturing. Future vaccine development must ensure that global equity is a core principle of coronavirus vaccine research and development (R&D) and that efforts are made in advance to anticipate and address issues that could impede this principle [21].

### 1.4 The role of an R&D roadmap

R&D roadmaps for diagnostics, therapeutics, and/or vaccines have been successfully developed and implemented for a number of pathogens and diseases. Examples include influenza, MERS-CoV, group A Streptococcus, bacterial meningitis, malaria, and *Mycobacterium tuberculosis* [22], [23], [24], [25], [26], [27], [28]. WHO is also in the process of finalizing R&D roadmaps for pathogens included in the WHO R&D Blueprint Initiative, which is aimed at preventing and controlling epidemics [29].

According to the WHO Generic Methodology for Developing and Implementing R&D Roadmaps for Priority Pathogens with Epidemic Potential (unpublished), “Roadmapping is applied where collaborative multi-partner efforts are required for R&D initiatives and product development activities cutting across several organizations; and for industry-wide R&D collaborations at the regional, national and global levels.” The development of broadly protective coronavirus vaccines clearly meets these criteria, as advancing an R&D agenda for these products will require ongoing global investment, and communication and coordination among researchers, funders, regulators, public-health policymakers, industry representatives, and multilateral and nongovernmental organizations. Given this need, the Center for Infectious Disease Research and Policy (CIDRAP) at the University of Minnesota, with funding from the Bill & Melinda Gates Foundation and The Rockefeller Foundation and with input from global experts and leaders in the field, led the development of the Coronavirus Vaccines R&D Roadmap (CVR) in 2022. The purpose of the CVR is to provide a framework and timeline to accelerate R&D of broadly protective coronavirus vaccines that are suitable globally for routine, prophylactic use or for stockpiling and emergency use if another novel coronavirus with pandemic potential emerges.

## 2 Methodology for roadmap development

The CIDRAP roadmap development team relied largely on experience gained from developing other R&D roadmaps, including the Influenza Vaccines R&D Roadmap [24], [25] and roadmaps developed for pathogens that are included in the WHO’s Blueprint to Prevent Epidemics (Ebola/Marburg, Lassa, Nipah, and Zika viruses) [29].

In early 2022, the CIDRAP team began conducting background research aimed at identifying gaps and barriers toward developing broadly protective coronavirus vaccines and organized this information into five topic areas. Once gaps and barriers were identified, the team drafted goals and milestones to address them. In April 2022, CIDRAP formed a project steering group of senior global leaders that included representatives from The Rockefeller Foundation; the Bill & Melinda Gates Foundation; the Wellcome Trust; the US National Institute of Allergy and Infectious Diseases, US National Institutes of Health; CEPI; several academic institutions (University of Iowa [USA], University of North Carolina [USA], Icahn School of Medicine at Mount Sinai [USA], and University of the Witwatersrand [South Africa]); and Biologics Consulting, a US-based consulting firm with expertise in regulatory issues. With input from the steering group, CIDRAP then established a CVR development taskforce of 39 international subject matter experts and global leaders who have diverse knowledge and experience in vaccine R&D and related topics. Taskforce members represent organizations based in nine countries in North America, Europe, Africa, Asia, and the Western Pacific. Taskforce and steering group members were organized into workgroups that aligned with the five topic areas; each workgroup convened virtually several times in 2022 to discuss roadmap drafts and provide expert input on roadmap content. In addition, CIDRAP consulted with several other experts, as needed, to clarify certain issues, particularly regarding policy, such as the current status of certain technology transfer efforts.

The roadmap sections were revised several times based on taskforce member input, and a draft was posted online on the CIDRAP website for public comment from October 24, 2022 to November 18, 2022. Availability of the roadmap for public comment was shared widely (via email and on social media) with a broad group of global stakeholders, including industry representatives. After public comments were incorporated, steering group and taskforce members reviewed the next version of the document and a final version was created based on that review.

## 3 Key issues for R&D of broadly protective coronavirus vaccines

The five sections of the CVR cover virology applicable to vaccine R&D, immunology and immune correlates of protection, vaccinology, animal and human infection models for coronavirus vaccine research, and policy and financing. Each section identifies barriers, gaps, strategic goals, milestones, and additional R&D priorities germane to that topic area. The goals are intended to be broad, whereas the milestones generally follow the SMART format (specific, measurable, achievable, realistic/relevant, and time-sensitive) [30]. The roadmap includes 20 goals and 86 milestones, 26 of which are ranked as high priority. The sections below summarize key highlights from each section; however, not all of the important issues are addressed in this report. The full version of the CVR can be found on the CIDRAP website [31], and all high-priority milestones are listed in Table 1.

**Table 1:** Coronavirus Vaccines R&D Roadmap: High-Priority Milestones by Topic Area and Strategic Goal.

**Virology Applicable to Vaccine R&D**	
**Strategic Goal 1.1**: Enhance and sustain the capacity to identify, characterize, and share SARS-CoV-2 variants of interest, concern, and high consequence among researchers globally.	• **Milestone 1.1.d**: By 2024, generate a financially sustainable collaborative international program to quickly identify, characterize, and share information on SARS-CoV-2 viruses, including antigenic information, in real time with the potential to build on current systems such as the WHO’s GISRS.
**Strategic Goal 1.2**: Improve characterization of the coronavirus universe to determine the diversity of strains necessary to ensure adequate breadth of coverage for vaccine R&D.	• **Milestone 1.2.c**: By 2024, devise a consensus approach to prioritize and select coronaviruses that would constitute an optimally diverse panel to be used in vaccine R&D for assessing breadth of protection. Selection criteria should initially focus on alphacoronaviruses and betacoronaviruses that: (1) use the hACE2 receptor, (2) grow in primary human cells, (3) are genetically diverse, (4) have been antigenically characterized, and (5) have strains available for study. • **Milestone 1.2.f**: By 2024, generate at least one initial panel of virus stocks featuring different coronaviruses and diverse cell lines that are readily susceptible to a wide range of coronaviruses, and make the panel accessible to researchers working on coronavirus vaccine R&D.
**Immunology and Immune Correlates of Protection**	
**Strategic Goal 2.1**: Ensure that clinical samples and immunoassays are available to the research community for improving understanding of the mechanisms of mucosal and systemic immunity related to SARS-CoV-2 infection.	• **Milestone 2.1.a**: By 2023, develop a centralized or virtual biorepository and an associated governance structure to use pre-COVID-19 pandemic clinical samples, including mucosal (e.g., nasal lavage and saliva) and serologic samples that are currently available from a range of research laboratories, potentially by tapping into existing biobanks. • **Milestone 2.1.c**: By 2024, establish and fund a centralized or virtual biorepository involving a new cohort of subjects from multiple regions of the world, to include those with a history of SARS-CoV-2 infection, for obtaining high-impact (e.g., mucosal, bronchoalveolar lavage, serologic, bone marrow) and appropriately collected and timed clinical samples. • **Milestone 2.1.d**: By 2024, develop new immunologic assays for SARS-CoV-2 research, as outlined in the plan identified in Milestone 2.1.b, and ensure that such assays are appropriately harmonized, standardized, and reproducible.
**Strategic Goal 2.2**: Define mechanisms of mucosal and systemic immunity relevant to SARS-CoV-2 infection and the development of broadly protective coronavirus vaccines.	• **Milestone 2.2.d**: By 2027, determine mucosal biomarkers, including systemic surrogates of mucosal immunity, that are predictive of mucosal immune protection against SARS-CoV-2 infection.
**Strategic Goal 2.3**: Clarify mechanisms for stimulating broadly protective mucosal and systemic immune responses that are cross-reactive for different coronaviruses.	• **Milestone 2.3.a**: By 2024, identify epitopes other than the RBD area of the S protein that generate protective humoral immunity and are conserved across different virus types.
**Strategic Goal 2.4**: Understand the mechanisms of durability of immune protection from SARS-CoV-2 and other coronaviruses.	• **Milestone 2.4.a**: By 2024, determine initial factors that influence duration of antibody and memory B- and T-cell responses following SARS-CoV-2 infection or vaccination, such as persistence of the germinal center.
**Strategic Goal 2.6:** Identify mechanistic correlates of protection for immunity generated by SARS-CoV-2 vaccines and broadly protective coronavirus vaccines.	• **Milestone 2.6.c**: By 2026, identify statistically validated CoPs for predicting the efficacy of SARS-CoV-2 vaccines based on key immune responses that correlate with specific clinical end points and that are applicable to viral variants with different characteristics. • **Milestone 2.6.d**: By 2027, determine one or more CoPs for mucosal vaccines against SARS-CoV-2 infection.
**Vaccinology**	
**Strategic Goal 3.1**: Define goals for broadly protective coronavirus vaccines by establishing a widely agreed upon and vetted set of PPCs and determine use cases for such vaccines.	• **Milestone 3.1.a**: By 2023, building on existing TPPs, develop a broadly agreed upon and internationally vetted (e.g., through a process involving an international multilateral organization such as the WHO) set of PPCs to identify key product characteristics, including optimal and critical minimal criteria. These could follow a tiered approach, with an initial focus on variant-proof SARS-CoV-2 vaccines, then moving to other, more broadly protective tiers.
**Strategic Goal 3.2**: Leverage new technologies or new approaches to create effective, durable, and safe vaccines that offer broad protection across different coronaviruses.	• **Milestone 3.2.b**: By 2023, publish the findings of a workshop on SARS-CoV2 mucosal vaccines to identify gaps in mucosal approaches for vaccine development. • **Milestone 3.2.d**: By 2024, develop and make available to researchers, an initial repository of coronaviruses (as available), pseudoviruses (if they can be made), and antigens. The repository could be developed in a tiered fashion, with an initial focus on the highest-risk viruses and then adding additional viruses over time. • **Milestone 3.2.i**: By 2027, further clarify, through clinical studies, if alternative routes of administration, including intranasal, transdermal, inhaled, and oral vaccines, can enhance mucosal immunity and protect against disease and transmission.
**Strategic Goal 3.4**: Build a foundation for regulatory evaluation of future coronavirus vaccines.	• **Milestone 3.4.b**: By 2023, develop a set of principles to inform regulatory evaluation of new coronavirus vaccines that outlines what information is required to demonstrate the need for and added value of variant-proof SARS-CoV-2 vaccines and to provide confidence in vaccine efficacy, particularly compared to approved vaccines. This set of principles would be less specific than regulatory guidance, since many details will still be unresolved, but such principles could be a valuable starting point for clarifying regulatory evaluation for such vaccines. • **Milestone 3.4.c:** By 2025 and building on Milestone 3.4.b, develop a set of principles for regulatory evaluation of future more broadly protective coronavirus vaccines that: (1) follows a tiered or stepwise approach (such as starting with predicting efficacy against sarbecoviruses other than SARS-CoV-2, then to merbecoviruses, and then to additional coronaviruses of concern as necessary); (2) takes into consideration the various mechanisms of protection that different vaccines may employ, which may inform the potential breadth of protection for a given vaccine construct; (3) clarifies what a “broadly protective coronavirus vaccine” means from a regulatory perspective and how breadth of protection is communicated in the associated product information; (4) identifies approaches for predicting protection provided by new vaccines (i.e., predicting potential clinical benefit) against coronaviruses that are not circulating in the human population; (5) defines the potential roles and limitations of tools such as animal studies, human infection studies, and immunobridging for predicting the breadth of protection for new vaccines; and (6) clarifies potential regulatory pathways for new coronavirus vaccines.
**Strategic Goal 3.5**: Facilitate the development of vaccine candidates with characteristics that meet global needs.	• **Milestone 3.5.c**: By 2027, support the development of coronavirus vaccine technologies that are suitable for broad access and global distribution (such as cold-chain–independent technologies) and that are scalable and can be produced affordably.
**Animal and Human Infection Models for Coronavirus Vaccine Research**	
**Strategic Goal 4.1**: Ensure that appropriate animal models are developed and available for conducting R&D for broadly protective coronavirus vaccines.	• **Milestone 4.1.a**: By 2024, develop a strategy to ensure that validated, reliable reagents, virus strains and stocks, and harmonized serological assays are available for studying a broader range of coronaviruses in animal models with initial focus on additional sarbecoviruses (group 2b betacoronaviruses) and a wider variety of MERS-related merbecoviruses (group 2c betacoronaviruses). • **Milestone 4.1.b**: By 2024, convene an international workshop on animal models for studying broadly protective coronavirus vaccines. Examples of topics for the workshop include: (1) review existing animal models for SARS-CoV, SARS-CoV-2, MERS-CoV, and other coronaviruses; (2) determine which animal models are best suited for R&D of broadly protective coronavirus vaccines; (3) identify strategies to optimize the use of small-animal models (e.g., mice, hamsters, ferrets); (4) determine how best to optimize and reduce the use of NHPs for R&D efforts, particularly given their limited supply; (5) determine how to mimic preexisting immunity in animal models; (6) determine how animal models can be used to assess the impact of host genomics or the microbiome on vaccine performance, such as through the use of “dirty mice”; (7) determine the role of animal models in measuring mucosal immunity, breadth, and durability of vaccines; (8) determine the role of animal models in defining immune CoPs; (9) determine the role of animal models in studying long COVID/PASC; (10) identify gaps in the current animal model landscape; and (11) develop strategies and plans for meeting future animal-model research needs. • **Milestone 4.1.d**: By 2025, ensure that standardized, validated, and well-characterized animal models are available to evaluate and compare broadly protective coronavirus vaccines. Examples of parameters to consider include the challenge virus strain; dose, route, volume, and timing of challenge; and animal responses to human-adapted variants. Immune history and prior exposure to ancestral coronaviruses should also be considered. The appropriate surrogate markers of clinical disease severity, such as weight loss or virus titers in the lungs, are needed for each animal species and for each virus sub-genus or relevant variant used to establish the model.
**Strategic Goal 4.2**: Establish the role of a CHIM in R&D for broadly protective coronavirus vaccines and optimize the model for vaccine research.	• **Milestone 4.2.c**: By 2024, develop a set of best practices for using a CHIM in coronavirus vaccine research to include risk mitigation strategies that reflect the changing landscape of disease and therapies. • **Milestone 4.2.d**: By 2025, establish parameters, in coordination with global regulators, for using CHIM studies and immunobridging for licensure of candidate vaccines. • **Milestone 4**.**2.e**: By 2025—assuming candidate vaccines are available—standardize parameters for a CHIM model in assessing broadly protective coronavirus vaccines, such as determining appropriate strain selection (which may need to be defined contextually at the time), standardizing panels of immunologic assays and assay harmonization, identifying mucosal inflammatory markers, and harmonizing protocols as possible.
**Policy and Financing**	
**Strategic Goal 5.1**: Establish and convey the value of sustained financial support and demand for development of broadly protective coronavirus vaccines.	• **Milestone 5.1.a**: By 2024, develop and disseminate a detailed economic case for broadly protective coronavirus vaccines through an FVVA or a series of detailed cost-benefit analyses for vaccines from SARS-CoV-2 variant-proof vaccines to more broadly protective coronavirus vaccines. These assessments will need to include a multitude of perspectives (e.g., health payers, economic, and societal) at a number of levels (e.g., global, national, and regional) and take into account varying contexts (e.g., demographics, healthcare capacity, competing health priorities) and the potential pathways for deployment, such as routine, preventive use or reactive outbreak control. • **Milestone 5.1.c**: By 2024, convene a meeting of vaccine investors, purchasers (including governments and large global institutions), producers, and governmental representatives aimed at exploring strategies for providing a reliable marketplace and financial model for broadly protective coronavirus vaccines. Meeting participants will assess the current push (e.g., grants, subsidies) and pull incentives (e.g., advance market commitments) and appropriate thresholds to move from push to pull, as well as establish a pricing model in line with the PPCs that can be anticipated for vaccines of various characteristics, such as the number of doses required, stability, duration of protection, and level of protection.

**Abbreviations:** CHIM, controlled human infection model; CoP, correlate of protection; FVVA, full value of vaccine assessment; GISRS, Global Influenza Surveillance and Response System; hACE2, human angiotensin converting enzyme-2; MERS-CoV, Middle East respiratory syndrome coronavirus; NHP, nonhuman primate; PASC, post-acute sequelae of SARS-CoV-2 infection; PPC, preferred product characteristics; RBD, receptor-binding domain; R&D, research and development; SARS-CoV, severe acute respiratory syndrome coronavirus; SARS-CoV-2, severe acute respiratory syndrome coronavirus 2; TPP, target product profile; WHO, World Health Organization.

**The milestones and goals identified in this table reflect only those that were ranked as high priority. They are organized by the order in which they appear in the Coronavirus Vaccines R&D Roadmap and reflect the numbering scheme of the roadmap. To see all goals and milestones, please refer to the complete roadmap.

### 3.1 Virology applicable to vaccine R&D

As noted above, implementing a tiered approach toward developing broadly protective coronavirus vaccines may be the most effective use of resources. With this model, a first tier is to ensure that next-generation coronavirus vaccines protect against all current and future variants of SARS-CoV-2 viruses. Global capacity to conduct surveillance and genomic sequencing for SARS-CoV-2 (particularly in LMICs) is critical for obtaining a more comprehensive and representative understanding of coronavirus distribution and evolution in humans, which can inform future vaccine development. However, disparities exist among countries and global regions in systems infrastructure, expertise, human and financial resources, and overall sequencing and surveillance capacity that constrain implementation of coordinated and uniform efforts to improve global SARS-CoV-2 genomic surveillance [32], [33]. Additionally, the lack of standardized and consistently applied nomenclature for variants complicates the interpretation and representativeness of available sequencing data for SARS-CoV-2 [32], [34].

Another critical issue for developing broadly protective coronavirus vaccines is the need to better characterize coronavirus diversity in animal reservoirs and to determine which viruses are most likely to spill over into human populations. This is essential to guide a coordinated, well-informed process of selecting diverse coronaviruses for vaccine R&D [20], [35]. While recent efforts have expanded coronavirus sampling of wild and captive animals, further work is needed to improve understanding of the geographic distribution, viral diversity, host range, prevalence, and spillover risks of these viruses and to link such information to human surveillance data and, ultimately, to vaccine R&D [20], [35], [36]. Generated viral sequencing data from such efforts should be open, accessible, and standardized (including metadata) to permit high-throughput analyses that could ultimately be used to bridge phylogenetic gaps present in the coronavirus virome and to determine the diversity that exists across different populations and geographic settings [20], [32], [35]. Since betacoronaviruses are currently considered to be at highest risk for spillover, research campaigns are particularly important for further characterizing this genus.

To address these and other issues, the virology section of the CVR includes four goals and 15 milestones that outline key activities, some of which are summarized as follows. (1) Generate a sustainable collaborative international program for quickly identifying, characterizing, and sharing genomic and antigenic information in real time on SARS-CoV-2 viruses identified in humans, potentially building on or integrating with what currently exists for influenza or other similar efforts, such as WHO’s Global Influenza Surveillance and Response System (GISRS) [37], [38], [39]. (2) Initiate research campaigns to identify diverse bat-derived and other animal coronaviruses (particularly group 2d betacoronaviruses) and to generate critical reagents needed to study such viruses. (3) Develop a coordinated international framework to enhance sampling of both wild and captive animals (particularly bats, and domesticated and companion animals) in geographically diverse regions for improving understanding of the distribution, viral diversity, host range, and prevalence of coronaviruses globally [20], [35], [36]. (4) Devise a consensus approach to prioritize and select coronaviruses that would comprise an optimally diverse panel to be used in vaccine R&D for assessing breadth of protection [35]. (5) Generate at least one initial panel of virus stocks featuring different coronaviruses and diverse cell lines that are readily susceptible to a wide range of coronaviruses and make the panel accessible to researchers working on coronavirus vaccine R&D [40], [41]. (7) Develop serologic platforms for conducting serosurveillance studies in high-risk human populations to identify signals suggesting the potential for spillover from animals to humans, as such efforts can be used to identify coronaviruses for vaccine R&D.

### 3.2 Immunology and immune correlates of protection

Generating broadly protective coronavirus vaccines requires greater understanding of a number of fundamental immunologic issues. First, efforts are needed to better characterize the mechanisms of mucosal and systemic immunity and the relative contributions of each in protecting against coronavirus disease, infection, and transmission [19], [42], [43], [44], [45]. To achieve this, a greater understanding of innate and adaptive immune responses to coronavirus infection in the various immune compartments (e.g., upper respiratory tract versus lung) is required [46], [47]. Second, research is needed to identify factors that influence breadth of protection against a diverse range of coronaviruses, such as identifying conserved epitopes [20], [35], [48], [49], [50], [51], [52], [53] and improving understanding of receptor-dependent antibody effector functions in developing cross-protection against multiple coronavirus strains [54], [55]. Third, researchers need to further explore how, and if, durable immunity (i.e., lasting at least one year or longer) can be generated by future coronavirus vaccine candidates. Owing to the short period of mucosal viral replication, natural infection may not be fully controlled by human immune responses, which creates challenges for developing durable vaccines [56]. Fourth, further clarification is needed regarding the role of preexisting immunity to SARS-CoV, SARS-CoV-2, and the “common-cold” coronaviruses on efficacy of future coronavirus vaccines, along with understanding the role of immune imprinting to initial coronavirus vaccine or infection exposures [57], [58], [59]. A final need is to identify correlates of protection (CoPs) for coronavirus vaccines based on different antigens, vaccine platforms, clinical outcomes (e.g., prevention of severe disease or infection), and modes of administration, including oral and intranasal vaccines aimed at stimulating mucosal immunity.

To address these complex issues, the immunology section of the CVR contains six strategic goals and 27 milestones. The first goal addresses the need for adequate clinical samples from centralized or virtual biorepositories involving existing and future human cohorts. It also recommends generating assays for using such samples and ensuring that assays are appropriately harmonized, standardized, and reproducible, as feasible. The second goal focuses on determining the relative roles of mucosal versus systemic humoral immunity in protecting against coronavirus infection and transmission [19], [42], [56]. For example, efforts are needed to define the initial cellular mechanisms of protection for SARS-CoV-2 infection at the mucosal surface, determine biomarkers that are predictive of mucosal immune protection, and develop a mucosal immunity “atlas” to collect and organize information on innate and adaptive coronavirus mucosal immunity that maps responses in different anatomic compartments (i.e., upper versus lower respiratory tract), and across different age-groups and geographic regions. The third goal focuses on issues related to improving breadth of protection, such as identifying B- and T-cell epitopes that generate protective humoral immunity and are conserved across different virus genera or subgenera, and identifying mechanisms underlying the induction of broadly protective immune responses. The fourth goal focuses on durability of protection and includes milestones aimed at determining initial factors that influence duration of antibody and memory B- and T-cell responses following SARS-CoV-2 infection or vaccination [59], [60], [61], [62], and identifying the determinants of longevity for antigen-specific plasma cells in bone marrow and in mucosa-associated lymphoid tissue [61]. The fifth goal addresses issues related to understanding the impact of preexisting immunity to SARS-CoV-2 or other coronaviruses, such as determining whether preexisting immunity causes antigenic superiority, how preexisting immunity affects recall responses, and how a primed immune system can be induced to generate broadly protective immune responses to divergent coronaviruses [57], [63]. The final goal includes milestones on creating a central database of existing CoPs for SARS-CoV-2 vaccines, identifying validated CoPs for predicting efficacy of next-generation (i.e., “variant proof”) SARS-CoV-2 vaccines, determining one or more CoPs for mucosal coronavirus vaccines [56], and determining whether multiple biomarkers are needed to increase the performance of a CoP for predicting vaccine efficacy for existing or broadly protective vaccines [64], [65], [66], [67], [68], [69].

### 3.3 Vaccinology

To inform future vaccine development priorities and strategies, a minimally acceptable target product profile (TPP) or set of preferred product characteristics (PPCs) is needed that focuses on broad protection as a starting point. Both PPCs and TPPs outline the preferential attributes for vaccines under development, but PPCs address early-stage research and are intended to promote innovation by providing broad guidance on development of new products or improvement of existing products [70]. TPPs are typically more specific and provide parameters that can inform R&D targets for funders and developers. Desirable attributes for either include not just vaccine efficacy, but also durability of protection, vaccine safety, manufacturing considerations, cold-chain requirements, and ease of distribution and use, particularly in LMICs.

Currently, a number of strategies for generating broadly protective coronavirus vaccines are under investigation, but additional long-term resources and investments are needed to further evaluate, incentivize, and advance vaccine candidates through the development pipeline, particularly into clinical trials. Several recent studies, for example, have found that a SARS-CoV-2 receptor-binding domain (RBD) and spike nanoparticle with an adjuvant elicited cross-neutralizing antibody responses against SARS-CoV, several SARS-CoV-2 variants, and several bat coronaviruses [51], [71]. Additionally, FcR-mediated cross-protective immune responses may be critically important in pan-sarbecovirus vaccine designs [54], [55]. Another approach is to generate vaccines that contain multiple representative immunogens from different virus strains, such as through development of chimeric spike vaccines or mosaic/multiplexed nanoparticle vaccines [50], [53], [72], [73], [74]. Prime-boost strategies using different immunogens is another possible mechanism for creating broadly protective vaccines [75].

In addition, a number of different platforms are under investigation, particularly for next-generation SARS-CoV-2 vaccines, such live-attenuated virus vaccines, whole inactivated virus vaccines, viral-vectored vaccines, recombinant protein subunit vaccines, peptide-based vaccines, virus-like particle and nanoparticle vaccines, and nucleic acid (deoxyribonucleic acid [DNA] or RNA) vaccines [76], [77], [78], [79]*.* Researchers still need to determine which platforms will provide the greatest breadth of protection and durability and demonstrate the ability to suppress immune-driven antigenic variation and emergence of vaccine escape mutants. Researchers also need to determine the role of different adjuvants for different vaccine antigen/platform combinations in improving immunogenicity of next-generation vaccines, including for use as a primary vaccine series versus boosting [80].

A key issue for evaluating vaccine candidates is to identify the best strategies for rapidly conducting randomized controlled trials of new vaccines in comparison to existing vaccines, as appropriate and depending on the regulatory pathway. Another key issue is to determine the best approaches for assessing efficacy of broadly protective coronavirus vaccines in naïve and preimmune populations and in populations with preexisting immunity from previous infection or vaccination [81], [82]. Additionally, broadly protective vaccines will likely need to show protection not only against circulating coronaviruses but also against viruses that are not circulating (i.e., “pre-emergent viruses”), which creates significant challenges for regulatory review and approval.

Three potential pathways exist for regulatory approval in the United States: traditional approval, which relies on efficacy data from randomized controlled trials; accelerated approval, which uses a surrogate marker to determine efficacy in clinical trials; and the US Food and Drug Administration’s (US FDA’s) Animal Rule, which relies on efficacy data from animal studies with immunobridging to humans [83], [84]. Human infection studies may also be used to define correlates of protection [85] or to demonstrate vaccine efficacy under certain situations [83]. Regulatory authorities in other countries have similar approaches, although they generally lack an animal rule option; however, animal data could still play a pivotal role in the benefit-risk assessment. The traditional approval pathway is the gold standard, but regulatory approval may be granted based on other pathways if the requirements of the traditional pathway cannot be met. To advance vaccine R&D, researchers need clarification regarding which pathways will be acceptable, particularly for vaccines that protect against coronaviruses not yet circulating in humans.

To address these issues, the vaccinology section of the CVR includes five strategic goals and 20 milestones. The first goal involves developing a broadly agreed upon and internationally vetted set of PPCs that builds on existing TPPs to identify key product characteristics, including critical minimal criteria and optimal criteria for broadly protective coronavirus vaccines, and generating use cases for such vaccines. The second goal involves leveraging new technologies or new approaches to create effective, durable vaccines that offer broad protection across different coronaviruses. Examples include identifying gaps in mucosal approaches for vaccine development; determining, primarily through preclinical studies, if any adjuvants can substantially improve vaccine efficacy, breadth, or durability for vaccines against SARS-CoV-2 variants or other coronaviruses; and conducting clinical studies to determine if intranasal, transdermal, or oral vaccines can enhance mucosal immunity and protect against both symptomatic disease and virus transmission [56]. Additionally, initial repositories of coronaviruses (as available), pseudoviruses (if they can be made), reagents, and antigens are needed for vaccine research. Another milestone involves defining a set of principles that can be used by funders and developers to down-select vaccine candidates for further evaluation. These should consider factors that influence the ability to use and manufacture vaccines in different regions of the world, such as complexity of manufacturing, challenges with vaccine distribution and use, and the end goals for using different vaccines. The third goal involves establishing principles for conducting clinical trials that allow for comparisons between vaccines, such as developing a set of harmonized clinical end points. The fourth goal focuses on building a foundation for regulatory evaluation of future coronavirus vaccines, including developing a set of principles for evaluation of next-generation, variant-proof SARS-CoV-2 vaccines and a set of principles for evaluation of broadly protective vaccines. The last goal advocates for the development of vaccine candidates suitable for global access and distribution (such as cold-chain—independent technologies) and that are useable, scalable, and affordable worldwide, particularly in LMICs.

### 3.4 Animal and human infection models for coronavirus vaccine research

This section addresses considerations for ensuring that a range of animal models are available for coronavirus vaccine R&D and that the controlled human infection model (CHIM) is optimized for assessing such vaccines. For animal models, an important issue is the need for multiple different models to assess vaccines that protect against different coronaviruses, particularly since not all coronaviruses bind to the same receptor [86], [87], [88], [89]. For example, SARS-CoV and SARS-CoV-2 bind to the hACE2 receptor (human angiotensin converting enzyme-2), but MERS-CoV binds to DPP4 (dipeptidyl peptidase 4), and the receptor site remains unknown for some of the viruses that cause milder disease in humans [90]. Animal models are also needed that recapitulate the range of clinical features of coronavirus infection found in humans and that can address the impact of host factors on vaccine efficacy [86]. For example, animal models are needed that are suitable for both antigenically naïve populations (i.e., infants and very young children) and antigenically experienced populations (i.e., people who have been infected with SARS-CoV-2 or vaccinated against the virus) [91], [92].

Recent experience with a CHIM for coronavirus vaccine research is limited, as so far only the United Kingdom has published reports using a CHIM for studying SARS-CoV-2 [93]. As such, clarification is needed regarding the role of CHIM studies for evaluating broadly protective coronavirus vaccines [83], [85], [94]. Issues that need to be addressed include standardizing parameters for CHIM research, developing best practices for using a CHIM in coronavirus vaccine R&D, determining the potential impact of prior infection or vaccination against SARS-CoV-2 on CHIM studies involving broadly protective coronavirus vaccines, and ensuring regulatory harmonization for conducting CHIM studies. Further clarification is also needed regarding how studies involving coronaviruses that cause mild disease in humans (human betacoronaviruses HKU1 and OC43 and human alphacoronaviruses 229E and NL63) could contribute to coronavirus vaccine R&D [20], [85].

The CVR includes two strategic goals under this topic: one for animal models, which includes eight milestones, and one for CHIM research, which includes seven milestones. For animal models, examples of important activities are summarized as follows. (1) Convene an international workshop on animal models for studying broadly protective coronavirus vaccines to review existing animal models for coronaviruses, identify gaps in the current animal model landscape, and recommend how to address those gaps. (2) Develop a strategy to ensure that validated, reliable reagents, highly heterogeneous and pathogenic virus strains and stocks, and harmonized serologic assays are available for studying a broad range of coronaviruses in animal models. (3) Ensure that standardized, validated, and well-characterized animal models are available to evaluate and compare broadly protective coronavirus vaccines against acute and chronic disease phenotypes. Examples of parameters to consider include the challenge virus strain; dose, route, volume, and timing of challenge; animal responses to human-adapted variants; and animal immune history.

For CHIM research, several important issues include the following. (1) Conduct a workshop to clarify the role of CHIM studies for evaluating broadly protective coronavirus vaccines and to develop consensus on how CHIM models can be used for coronavirus vaccine research [94]. (2) Develop a set of best practices for using a CHIM in coronavirus vaccine research, to include risk-mitigation strategies [85]. Prior experience with CHIM models for other viral respiratory pathogens, such as influenza virus and respiratory syncytial virus can inform this activity [95], [96]. (3) Establish parameters, in coordination with global regulators, for using CHIM studies and immunobridging for licensure of candidate vaccines, which has been considered for other pathogens [97]. (4) Establish international capacity and collaborative networks for conducting CHIM studies of broadly protective coronavirus vaccines; this should include ensuring availability of contemporary, non-tissue culture-adapted challenge viruses and immune assays.

### 3.5 Policy and financing

Multiple barriers exist in bringing broadly protective coronavirus vaccines to market. First, since the emergency phase of the COVID-19 pandemic has largely passed, political will and public support for large-scale investments is diminishing [21], [98]. Second, companies face high opportunity costs in developing new vaccines; therefore, unless significant problems emerge with current vaccines, little incentive exists to invest in next-generation vaccines [99]. Third, maximizing the potential benefit of vaccination relies on global demand and vaccine uptake, which are uncertain for broadly protective coronavirus vaccines. Finally, intellectual property rights can pose a significant hurdle. Officials in the public sector are reluctant to increase public investment when they are unclear if there will be commensurate public access to intellectual property established through the use of public funds [100]. The role of patent pools, such as WHO’s COVID-19 Technology Access Pool (C-TAP), and the role of vaccines capitalizing on established technologies that are not patent-protected also require further clarification [101], [102].

Ensuring global equity in vaccine access will need to address the geographic concentration of vaccine R&D, manufacturing, and purchasing power of high-income countries (HICs), which can lead to gross inequities in vaccine distribution. A global concentration of manufacturing and regulatory capacity exists in HICs and in some countries with very large populations, guaranteeing them a large national market. Successful technology transfer to other countries or regions is complex and requires trusted partners with the expertise and capacity, long-term human and financial investment, and political will. Manufacturing capacity is not merely an issue of building the facilities and expertise, but also having the ability to maintain capacity in a financially sustainable way over time, particularly during non-pandemic times.

To address policy and financing issues for coronavirus vaccines, the CVR contains three strategic goals and nine milestones. The first goal involves establishing and conveying the value of sustained financial support and demand for development of broadly protective coronavirus vaccines. One key milestone under this goal is to develop and disseminate a full value of vaccine assessment (FVVA) or a series of detailed cost-benefit analyses for vaccines—from SARS-CoV-2 variant-proof vaccines to more broadly protective coronavirus vaccines [103]. Another important milestone is to convene a meeting of vaccine investors, purchasers (including governments and large global institutions), producers, governmental representatives, and others aimed at exploring strategies for providing a reliable marketplace and financial model for developing and producing broadly protective coronavirus vaccines. The second goal involves reassessing the current landscape of intellectual property rights to improve information sharing involving new technologies. The third goal is aimed at building a sustainable and more balanced geographic distribution of manufacturing capacity over time with expertise to manufacture high-quality vaccines for local use.

## 4 Conclusion

Global interest and investment in SARS-CoV-2 vaccine R&D has waned substantially since crisis-oriented initiatives have ended. Resources, therefore, for developing broadly protective coronaviruses will likely be insufficient in the coming years to generate broadly protective coronavirus vaccines, owing to shifting priorities of governments and other funding organizations. This roadmap outlines a framework for moving these vaccines forward, but without dedicated long-term resources or a coordinated governance structure to advance the activities outlined in the roadmap, the global R&D community may not be able to generate these vaccines in a timely manner. We must not lose sight of the fact that spillover events from animal reservoirs are becoming more prevalent because of increased interactions between humans and wild animals, such as land-use changes, disruption of natural ecosystems, increased urbanization, travel, climate change, and wildlife trade and consumption [104], [105]. The 21st century has already experienced the emergence of three human coronaviruses, ongoing avian influenza outbreaks and influenza epidemics, repeated filovirus outbreaks, and major flavivirus epidemics (such as Zika), resulting in trillions of dollars in economic costs and losses globally. Thus, we must use the recent experience with COVID-19 as a catalyst for changing existing paradigms and as an opportunity to look forward toward enhancing global health security and pandemic preparedness.

### CRediT authorship contribution statement

**Kristine A. Moore:** Conceptualization, Writing – original draft, Writing – review & editing. **Tabitha Leighton:** Conceptualization, Writing – review & editing. **Julia T. Ostrowsky:** Conceptualization, Writing – review & editing. **Cory J. Anderson:** Conceptualization, Writing – review & editing. **Richard N. Danila:** Conceptualization, Writing – review & editing. **Angela K. Ulrich:** Conceptualization, Writing – review & editing. **Eve M. Lackritz:** Conceptualization, Writing – review & editing. **Angela J. Mehr:** Conceptualization, Writing – review & editing. **Ralph S. Baric:** Writing – review & editing. **Norman W. Baylor:** Writing – review & editing. **Bruce G. Gellin:** Writing – review & editing. **Jennifer L. Gordon:** Writing – review & editing. **Florian Krammer:** Writing – review & editing. **Stanley Perlman:** Writing – review & editing. **Helen V. Rees:** Writing – review & editing. **Melanie Saville:** Writing – review & editing. **Charlotte L. Weller:** Writing – review & editing. **Michael T. Osterholm:** Writing – review & editing. **The Coronavirus Vaccines R&D Roadmap Taskforce:** Analysis and Interpretation, Writing – review and editing.

## Declaration of Competing Interest

The authors declare the following financial interests/personal relationships which may be considered as potential competing interests: [Galit Alter: Employed by Moderna. Dan Barouch: Co-inventor on vaccine patents that have been licensed to Janssen. Ralph Baric: Holds IP on sarbecovirus universal vaccine design, on the SAB for VacArt, and has collaborated with Adiago and NIH on Moderna mRNA vaccines. Norman Baylor: Provides regulatory advice and strategy to the regulated biopharma industry. Luciana Borio: In addition to holding a position at the Council on Foreign Relations, is a venture partner at Arch Venture Partners, an early-stage life sciences and biotechnology venture firm. Rachel Chikwamba: Has a grant with the Bill & Melinda Gates Foundation on localizing biologics manufacturing in South Africa. Cheryl Cohen: Has received grant support from Sanofi Pasteur, the US Centers for Disease Control and Prevention (CDC), Wellcome Trust, the Programme for Applied Technologies in Health (PATH), the Bill & Melinda Gates Foundation, and the South African Medical Research Council (SA-MRC). Bruce Gellin: Works for The Rockefeller Foundation, which is one of the funders of this work. Jonathan Heeney: Funded by CEPI and the Bill & Melinda Gates Foundation/Flu Lab to develop pre-pandemic vaccines for coronaviruses and influenza, respectively. Florian Krammer: The Icahn School of Medicine at Mount Sinai has filed patent applications relating to SARS-CoV-2 serological assays (US Provisional Application Numbers: 62/994,252, 63/018,457, 63/020,503 and 63/024,436) and NDV-based SARS-CoV-2 vaccines (US Provisional Application Number: 63/251,020) which list FK as co-inventor. Patent applications were submitted by the Icahn School of Medicine at Mount Sinai. Mount Sinai is seeking to commercialize a mucosal NDV-based SARS-CoV-2 vaccine; therefore, the institution and its faculty inventors could benefit financially. Mount Sinai has spun out a company, Kantaro, to market serological tests for SARS-CoV-2. FK has consulted for Merck, Seqirus, Curevac, and Pfizer, and is currently consulting for Pfizer, Third Rock Ventures, Merck, and Avimex. The FK laboratory is also collaborating with Pfizer on animal models for SARS-CoV-2. Teresa Lambe: Vaccine Taskforce via NIHR Support: Grant to support the running of the trial paid to University of Oxford. AstraZeneca Support: Support for medical writing. Vaccitech Consultant Fees for an unrelated project. Seqirus honoraria: Meeting relating to influenza meeting—unrelated work. Named as an inventor on a patent application for a vaccine against SARS CoV-2. Jason McLellan: Is an inventor on patents and patent applications regarding coronavirus vaccines and antibodies. Angela Mehr: Holds small amount of shares in AstraZeneca and Moderna. Kayvon Modjarrad: Current affiliation is as an employee of Pfizer, Inc. Peter Openshaw: Has participated in scientific advisory boards for GSK, Moderna, Janssen, Seqirus, and Pfizer. Peter Paradiso: Consultant to Pfizer, Member of Board of Directors at Dynavax. Stanley Plotkin: Consultant to Moderna, Sanofi, Merck, Janssen, Inovio, NTx Bio, Codagenix, Vaxinnity, Valneva, Meissa, and Rational. Gregory Poland: Offers consultative advice on COVID-19 vaccine development to AstraZeneca, Pfizer, Medicago, Johnson&Johnson/Janssen, Novavax, and Moderna. GAP has received grant funding from ICW Ventures for preclinical studies on a peptide-based COVID-19 vaccine for which he holds a patent. These activities have been reviewed by the Mayo Clinic Conflict of Interest Review Board and are conducted in compliance with Mayo Clinic Conflict of Interest policies. Andrew Pollard: Oxford University has an agreement with AstraZeneca for development of a COVID-19 vaccine. AJP led the clinical development of the Oxford-AstraZeneca vaccine. He is chair of the UK Government's Joint Committee on Vaccination and Immunisation, but does not participate in the COVID-19 committee. Melanie Saville: Has shares with Sanofi, a vaccine company; is an employee of CEPI, an organization that funds SARS CoV 2 and broadly protective coronavirus vaccine development. Lin-fa Wang: Co-inventor of patents on test, vaccine, and monoclonal antibodies for SARS-related coronaviruses. Daniela Weiskopf: The La Jolla Institute for Immunology has filed for patent protection for various aspects of T-cell epitope and vaccine design work. E. John Wherry: Is a member of the Parker Institute for Cancer Immunotherapy. EJW is an advisor for Danger Bio, Janssen, Merck, Marengo, New Limit, Pluto Immunotherapeutics, Related Sciences, Santa Ana Bio, Synthekine, and Surface Oncology. EJW is a founder of and holds stock in Surface Oncology, Danger Bio, and Arsenal Biosciences. Michael Worobey: Has received consulting fees on SARS-CoV-2 and the COVID-19 pandemic.]

## Data Availability

No data was used for the research described in the article.
